# Customer segmentation in the digital marketing using a *Q*-learning based differential evolution algorithm integrated with *K*-means clustering

**DOI:** 10.1371/journal.pone.0318519

**Published:** 2025-02-07

**Authors:** Guanqun Wang

**Affiliations:** 1 College of Accounting, Ningbo University of Finance & Economics, Ningbo, China; 2 Zhejiang Marine Development Think Tank Alliance, Ningbo, Zhejiang, China; Aalto University, FINLAND

## Abstract

Effective and well-structured customer segmentation enables organizations to accurately identify and comprehend the distinct characteristics and needs of various customer groups, thereby facilitating the development of more targeted marketing strategies. Contemporary artificial intelligence technologies have emerged as the predominant tools for customer segmentation, owing to their robust capabilities in analyzing complex datasets and extracting profound customer insights. This paper proposes a customer segmentation framework within the realm of digital marketing, which integrates a reinforcement learning-based differential evolution algorithm with *K*-means clustering using dimensionality reduction techniques to address challenges in the customer segmentation process. Initially, a correlation matrix is used to identify redundant noise and multicollinear features within customer feature groups, and Principal Component Analysis is applied for denoising and dimensionality reduction to enhance the ability of the model to identify potential features. Subsequently, a parameter adaptive adjustment method based on *Q*-learning is proposed, which significantly augments the clustering performance of *K*-means. Ultimately, the effectiveness of the proposed method is validated using a Kaggle dataset, and the elbow method is employed to ascertain the optimal number of clusters. Based on the cluster category centers, the typical characteristics of different customer types are analyzed. Furthermore, four widely recognized machine learning methods are employed to classify the clustering results, achieving over 95% classification accuracy on the test set. The experimental results demonstrate that the proposed model exhibits a high degree of customer characteristic identification and segmentation, which not only enhances marketing efficiency and customer satisfaction but also fosters corporate profit growth through the strategic formulation of various marketing initiatives.

## 1. Introduction

With the rapid advancement of network information technology, the digital economy has progressively established a significant presence within the traditional economic landscape [1, 2]. This digital economy embodies a new economic paradigm primarily driven by digital knowledge and information [3, 4]. Digital marketing constitutes a fundamental and integral component of the digital economy. By leveraging internet-based operational models, enterprises can effectively reach their target markets and optimize customer experiences, thereby facilitating sales and revenue growth [5–7]. In this context, marketing capabilities within the digital economy have emerged as a crucial factor in ensuring and enhancing corporate revenue, serving as a vital strategy for companies to maintain their competitive advantages in the market [8, 9]. Understanding customer characteristics and segmenting these characteristics based on similarities is a key approach for enterprises to achieve efficient digital marketing. Within the digital economy, customer segmentation refers to the process of categorizing customer attributes—such as demographic, behavioral, and geographic—obtained through online channels into distinct sub-clusters [10]. This segmentation enables companies to assess the loyalty of various customer groups and develop tailored marketing strategies aimed at maximizing business revenue [11]. By comprehending the unique needs and preferences of each segment, businesses can optimize their marketing efforts and enhance customer engagement, ultimately driving greater profitability [12].

Customer segmentation presents a significant challenge for businesses [13–15]. Firstly, customer data is often sourced from multiple channels, which complicates the integration of this data into a cohesive and comprehensive customer profile [16]. Furthermore, the data collected is frequently characterized by inaccuracies and gaps, which can severely undermine the effectiveness of the segmentation process [17]. Secondly, customer preferences and behaviors are not static; they evolve in response to dynamic market conditions [18]. Fluctuations in the external market environment and emerging trends can significantly influence customer needs and behavioral characteristics [19]. Moreover, effective customer segmentation typically necessitates the use of sophisticated data analysis tools and methodologies [20]. While advancements in artificial intelligence, particularly through machine learning techniques, have enhanced the analytical capabilities of organizations to some extent, existing methods often exhibit various limitations in practical application. This underscores the need for a more comprehensive analytical approach to thoroughly understand customer behavioral characteristics, thereby facilitating complete and effective customer segmentation [21–24].

To address the challenges encountered in the customer segmentation process, this paper proposes a customer segmentation framework that integrates a reinforcement learning-based differential evolution algorithm with *K*-means clustering in a dimensionality reduction context. First, a correlation matrix is employed to analyze redundant noise and multicollinear features within the dataset. Subsequently, Principal Component Analysis (PCA) is applied to eliminate redundant noise from the feature data, resulting in a set of low-dimensional, uncorrelated principal component features that enable the classification model to effectively identify potential features within the data. Second, to overcome the limitations of the traditional differential evolution algorithm, which utilizes a fixed scaling factor that does not adapt dynamically to varying problems, a parameter adaptive adjustment method based on *Q*-learning is proposed. This method is effectively integrated with the *K*-means clustering algorithm, significantly enhancing the clustering performance of *K*-means. To validate the effectiveness of the proposed method, we utilize the Kaggle dataset for empirical testing, employing the elbow method to determine the optimal number of clusters for the algorithm. The typical features of different customer types are then delineated based on the cluster centers. Finally, we apply four popular machine learning methods to classify the clustering results, thereby assessing the practicality of the clustering outcomes. The experimental results demonstrate that all four methods achieve over 95% classification accuracy on the test set. We recommend the use of Artificial Neural Networks (ANN) and Kernel Support Vector Machines (SVM) for classifying new customers.

In summary, the main contributions of this study are as follows:

- A novel customer segmentation framework is introduced that enhances segmentation quality by reducing feature dimensions and effectively integrating a reinforcement learning-based differential evolution algorithm with the *K*-means algorithm.
- A *Q*-learning-based adaptive dynamic adjustment method for the differential scaling factor is presented, allowing for improved adaptability to diverse problem environments and yielding superior search results.
- The elbow method is employed to determine the optimal number of clusters for customer segmentation. Additionally, four widely used machine learning techniques are utilized to assess the classification accuracy of the clustering results, underscoring the practical applicability of the proposed customer segmentation approach.

The remainder of the work is organized as follows. **Section 2** gives the state-of-the-art work on customer segmentation; **Section 3** proposes customer feature dimensionality reduction and combined clustering methods; The experimental results and analysis are presented in **Section 4**; **Section 5** summarizes the findings and outlines potential future work.

## 2. Related work

Customer segmentation plays a crucial role in marketing and data analysis [25–27]. The RFM (Recency, Frequency, Monetary) segmentation method is a widely used technique in marketing and customer relationship management [28]. The RFM model divides customers into different groups by analyzing their purchasing behavior in order to formulate more targeted marketing strategies [29, 30]. Christy et al. [31] employed RFM analysis to conduct customer segmentation and subsequently enhanced this approach by making minor modifications to the existing *K*-means clustering algorithm. Their findings indicated that the proposed algorithm outperformed other methods in terms of effectiveness. However, it is noteworthy that the study did not take into account the performance of customers within each segment. Rungruang et al. [32] proposed an innovative clustering algorithm that integrates both implicit and explicit knowledge by combining RFM analysis with formal concept analysis (FCA). Experimental results demonstrate that this proposed method can effectively generate practical marketing strategies for real-world businesses. However, the extraction and interpretation of implicit knowledge within the model may be influenced by data quality and completeness, which could limit the generalizability and applicability of the approach. The effectiveness of the RFM model is highly dependent on the quality and integrity of the data. If the data is inaccurate or incomplete, it may lead to incorrect customer classification and inappropriate marketing strategies [33, 34].

With the rapid development of computer science, AI technologies, particularly those represented by machine learning and deep learning, are increasingly being applied to the field of customer segmentation [35–37]. Yadegaridehkordi et al. [38] conducted a comprehensive study in which they first employed the *K*-means clustering algorithm to segment online reviews from travelers. Following this segmentation, they applied the Technique for Order Preference by Similarity to Ideal Solution (TOPSIS) method [39] to prioritize green hotel attributes based on their importance within each identified segment. To further enhance their analysis, they utilized the Classification and Regression Trees (CART) method [40] to investigate the relationship between environmentally friendly hotel characteristics and traveler satisfaction. This integrated approach provides managers with valuable insights for developing and implementing environmentally sustainable practices in the hospitality industry. Nilashi et al. [41] developed a new method for customer segmentation and preference prediction using text mining and predictive learning techniques and adopted clustering technology for customer segmentation. The effectiveness of the proposed method was evaluated using a restaurant dataset. The experimental results showed that the proposed method can better reveal customer satisfaction and make high-precision predictions of their preferences through their purchasing behavior. Wang et al. [10] employed the RFM model to preprocess the data and utilized an improved social spider optimization (MSSO) technique to select relevant customer features. They then applied a self-organizing neural network to identify six key features for clustering, which facilitated the formation of distinct customer segments. Finally, the Deep Neural Network (DNN) method [42] was used for customer segmentation. The experimental results indicate that the segmentation outcomes of the proposed approach surpass those of traditional methods, offering significant reference value for digital marketing strategies. Alkhayrat et al. [43] proposed a customer segmentation method that integrates Principal Component Analysis (PCA) for dimensionality reduction with an autoencoder neural network. The method reduces the feature space of original data before applying the *K*-means clustering algorithm for evaluation. Simulation experiments conducted on real telecommunications datasets demonstrate effective utilization of clustering in both reduced and latent spaces. This a dual strategy enables a deeper understanding of customer preferences and needs, ultimately leading to higher quality clustering results.

The analysis indicates that integrating traditional RFM models with AI-based technologies can effectively address the dynamic requirements of customer segmentation, reducing the risks associated with incorrect segmentation and decision-making that may arise from reliance on conventional methods. Despite the availability of numerous technical approaches for customer segmentation and the achievements realized through these methods, the increasing complexity of data sources and formats poses significant challenges, as existing technologies often encounter difficulties in processing and analyzing this diverse data effectively. Combining heuristic algorithms with AI classification models has proven to be a reasonable and effective approach [44–47]. Consequently, the adoption of customer segmentation models that utilize heuristic algorithms and artificial intelligence technologies is recommended, as these methodologies can provide deeper insights into customer behavior and preferences, thereby enabling organizations to make more informed decisions and adapt strategies to meet the evolving demands of the market. This approach not only enhances the accuracy of customer segmentation but also aligns with the necessity for businesses to remain agile in a rapidly changing environment.

## 3. Proposed methodology

### 3.1 Feature dimension reduction strategy based on PCA

**Fig 1:**
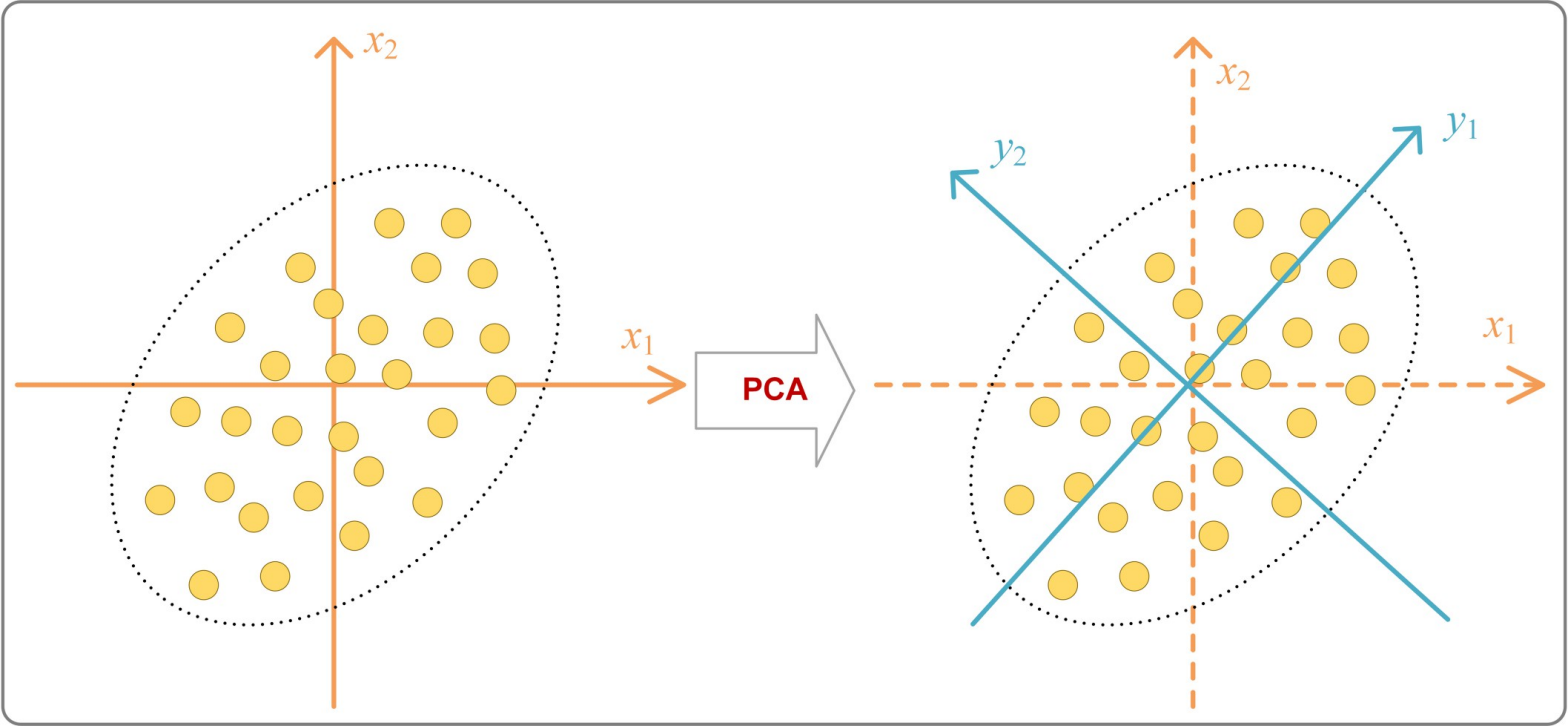
Schematic diagram of PCA principle.

Customer segmentation data often exhibits high correlations among features, along with the presence of irrelevant features, which significantly increases the difficulty of precise identification and classification by modeling techniques [48]. Principal Component Analysis (PCA) [49], illustrated in Fig 1, is a widely used unsupervised dimensionality reduction technique that employs orthogonal transformation to convert multiple linearly correlated variables into a smaller number of linearly independent principal components.

In the dimensionality reduction process of principal component analysis (PCA), one of the key steps is to select the first few principal components that can explain the largest possible variance in the data [50]. In practical implementation, PCA begins with the normalization of the data, ensuring that each feature contributes equally to the analysis. Following this, the covariance matrix of the dataset is computed. The next step involves performing an eigenvalue decomposition of the covariance matrix to extract the eigenvectors and eigenvalues. Here, the eigenvectors represent the basis of the new feature space, while the eigenvalues indicate the importance of each principal component in terms of the variance it explains. Finally, the number of principal components to retain is determined by examining the cumulative variance explained by the principal components. A common practice is to select enough components to reach a specified threshold of explained variance, ensuring that the selected components capture a significant portion of the data’s variability. Through these steps, PCA effectively reduces the dimensionality of the data while preserving as much relevant information as possible, facilitating subsequent data analysis and modeling tasks.

### 3.2 The basic *K*-means clustering algorithm

**Fig 2:**
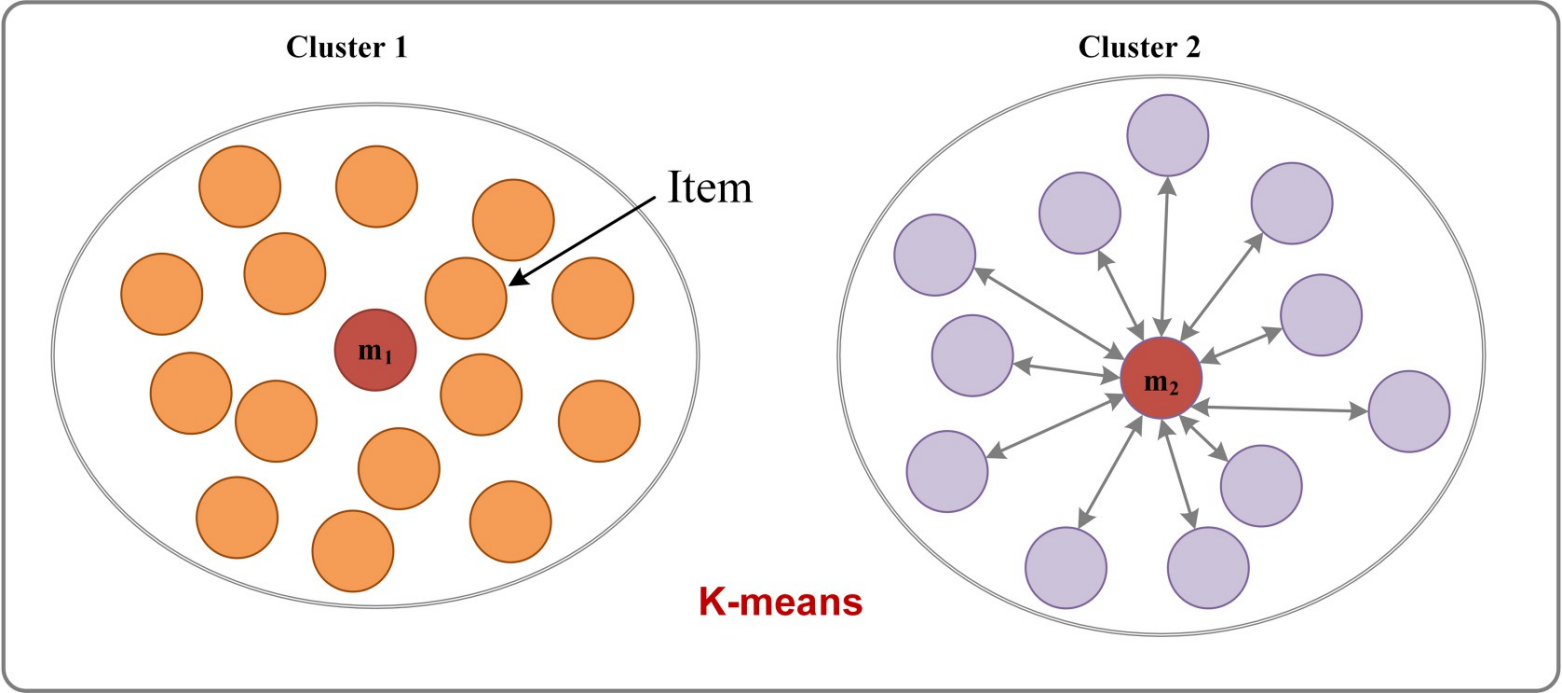
Schematic diagram of *K*-means algorithm.

The *K*-means clustering algorithm [51], illustrated in Fig 2, is an unsupervised hard clustering method that partitions a sample set. Given a dataset ***X***_*o*_ = {*x*_1_, *x*_2_, *x*_3_,…, *x*_*N*_} consisting of *N* samples, where each sample is characterized by *d* features, the distance between any two samples ***x***_*i*_ and ***x***_*j*_ can be mathematically represented using a distance metric, defined as follows:

$$d(x_{i},x_{j})=\sum_{l=1}^{d}{\left(x_{il}-x_{kl}\right)}^{2}={\left\|x_{i}-x_{j}\right\|}^{2}$$ (1)

where *d*(***x***_*i*_, ***x***_*j*_) represents the squared Euclidean distance; *x*_*il*_ represents the value of the *i-*th sample with respect to the *l*-th feature.

The *K*-means clustering method typically involves identifying *K* cluster centers C (where C ∈ {*m*_1_, *m*_2_, …, *m*_*k*_}) that partition the samples in the dataset ***X***_*o*_ so that the sum of squared Euclidean distance (SSE) [52] is minimized:

$$\min\;SSE=\sum_{l=1}^{K}\sum_{C(i)=l}\|x_{i}-m_{l}{\|}^{2}$$ (2)

where ***m***_*l*_ represents the center position vector of the *l*-th cluster, defined as follows:

$$m_{l}=\frac{1}{n_{l}}\sum_{C(i)=l}x_{i},\;l\in \{1,\cdots ,k\}$$ (3)

where *n*_*l*_ represents the number of samples in the *l*-th cluster partition. In addition, the optimal solution for *K*-means clustering is an NP-hard problem and is typically addressed using an iterative method.

### 3.3 The differential evolution algorithm based *Q*-Learning (QLDE)

#### A. The differential evolution algorithm

**Fig 3:**
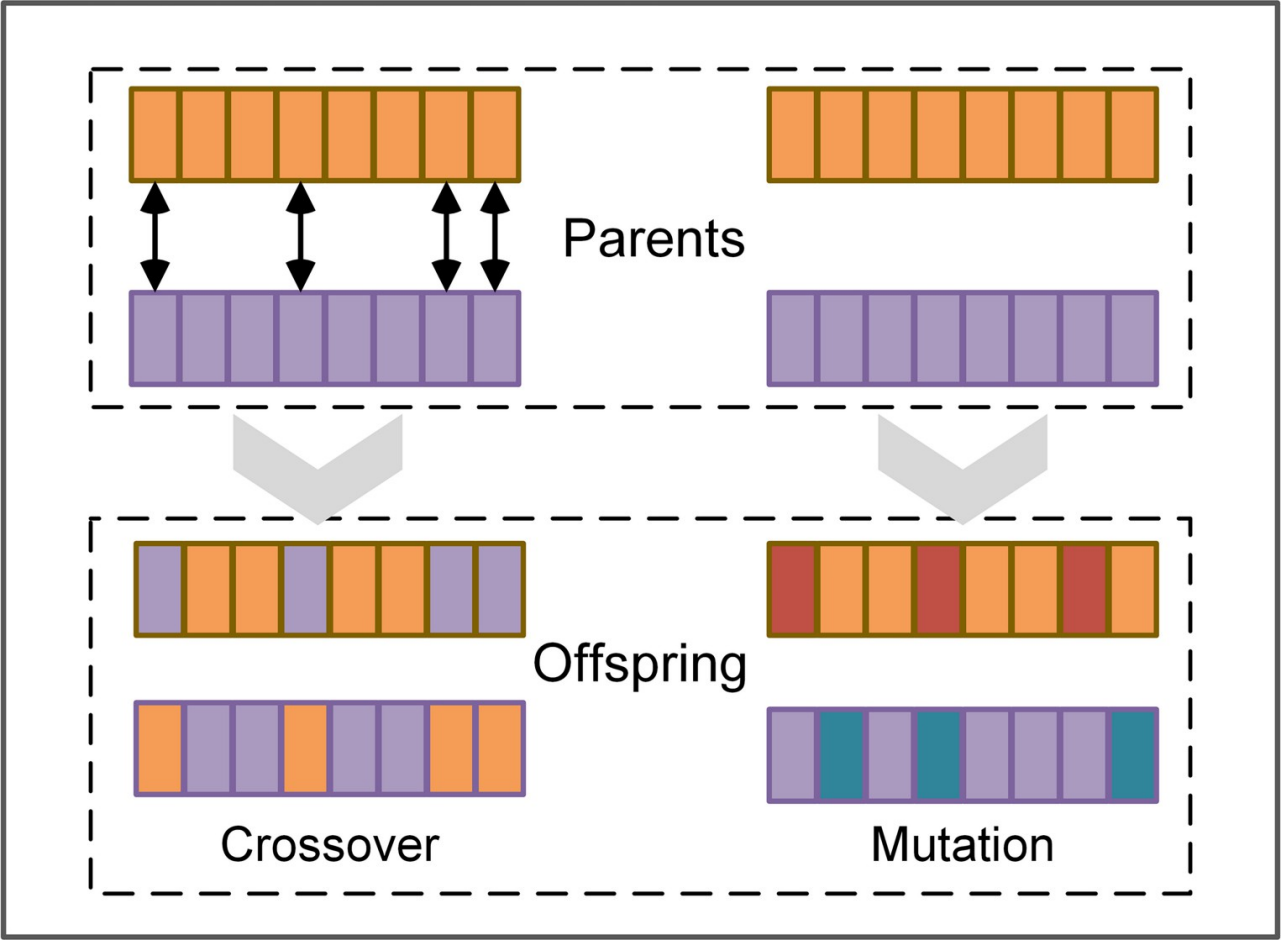
Crossover and mutation process of DE.

The Differential Evolution (DE) algorithm [53], is a well-known heuristic optimization technique inspired by the principles of population evolution. It has been successfully applied across various fields, including engineering [54], machine learning [55], and operations research [56], yielding compelling results. The DE algorithm primarily consists of four key processes: initialization, mutation, crossover, and selection. While the mutation and crossover processes are illustrated in Fig 3. And four key processes are described as follows:

1. Initialization process
  The initial generation method of the population will affect the performance of the algorithm. To ensure the diversity of the algorithm in the early stage, Logistic mapping [57] is used to generate chaotic sequences and generate the initial population, Logistic mapping is showed as follows:
$$\phi_{j+1}=\mu \cdot \phi_{j}\cdot (1-\phi_{j})$$ (4)
  where *ϕ*_*j*_ a represents the chaotic random number generated in the *j*-th dimension of the problem, when *j* = 0, *ϕ*_*j*_ is a random number in [0, 1]; *μ* is the control parameter, typically chosen within the range [3.57, 4], then the generation method of the population can be expressed as:
$$z_{ij}^{k}=LB_{j}+\phi_{j}\cdot (UB_{j}-LB_{j})$$ (5)
  where $z_{ij}^{k}$ represents the position of the *j*-th dimension of *i*-th individual at *k-*th iteration; *LB*_*j*_ and *UB*_*j*_ are represent the lower and upper boundary limits of the problem in the *j*-th dimension, respectively.
2. Cluster-guided mutation process
  The mutation process generates a new parameter vector by adding a weighted difference vector, derived from any two individuals in the population, to a third individual. However, traditional mutation operations primarily ensure the randomness of the algorithm and do not provide a reliable guiding direction. To address this limitation, this paper introduces a clustering strategy into the population. Specifically, the top 50% of individuals with better fitness values in each generation are considered to have a better guiding direction, and then they are combined with differential vectors to form new mutated individuals. This approach facilitates the exploration of new solution spaces and enhances the ability of population to adapt to various characteristics of objective functions by incorporating randomness into the search process. The details are shown as follows:
$$v_{i}^{k}=z_{Lk}^{k}+F\cdot (z_{i1}^{k}-z_{i2}^{k})$$ (6)
  where $v_{i}^{k}$ represents the mutation position vector corresponding to the *i*-th individual at the *k*-th iteration; *F* is the scaling factor, which controls the range of mutation; *Lk* denotes the position index randomly selected from the top 50% of the entire population with better performance at the *k*-th iteration. *i*1 and *i*2 are the index of a position randomly selected from the entire population at the *k*-th iteration.
3. Crossover and selection process
  The crossover process primarily involves combining the mutation vector with the current individual to generate a trial vector. This process is characterized by the crossover factor *C*_*r*_, which indicates the degree of information exchange. A larger *C*_*r*_ value suggests that the algorithm can explore a broader range of positions, while a smaller *C*_*r*_ value may lead to premature convergence, which is detrimental to global optimization. The selection process evaluates the fitness of the current individual and the trial vector. Following the principle of survival of the fittest, individuals with better fitness performance are carried forward into the next iteration of the cycle, as follows:
$$u_{ij}^{k}=\{\begin{array}{l} z_{ij}^{k},if[r_{2}(0,1)\leq C_{r}]or[j=j_{rand}]\\ v_{ij}^{k},else \end{array},j=\{1,2,\ldots ,J\}$$ (7)
$$z_{i}^{k+1}=\{\begin{array}{l} z_{i}^{k},if[f(z_{i}^{k})<f(u_{i}^{k})]\\ u_{i}^{k},else \end{array}$$ (8)
  where $u_{ij}^{k}$ represents the position of the *j*-th dimension of *i*-th trial individual at *k-*th iteration; *r*_2_(0,1) is the random number in the range [0, 1]; *C*_*r*_ is the crossover factor; *j*_*rand*_ represents the dimension index randomly selected from the total dimension *J* of the individual; *f*($z_{i}^{k}$) and *f*($u_{i}^{k}$) are the fitness value of the *i*-th position vector $z_{i}^{k}$ and trial vector $u_{i}^{k}$, respectively.

#### B. The *Q*-learning algorithm

**Fig 4:**
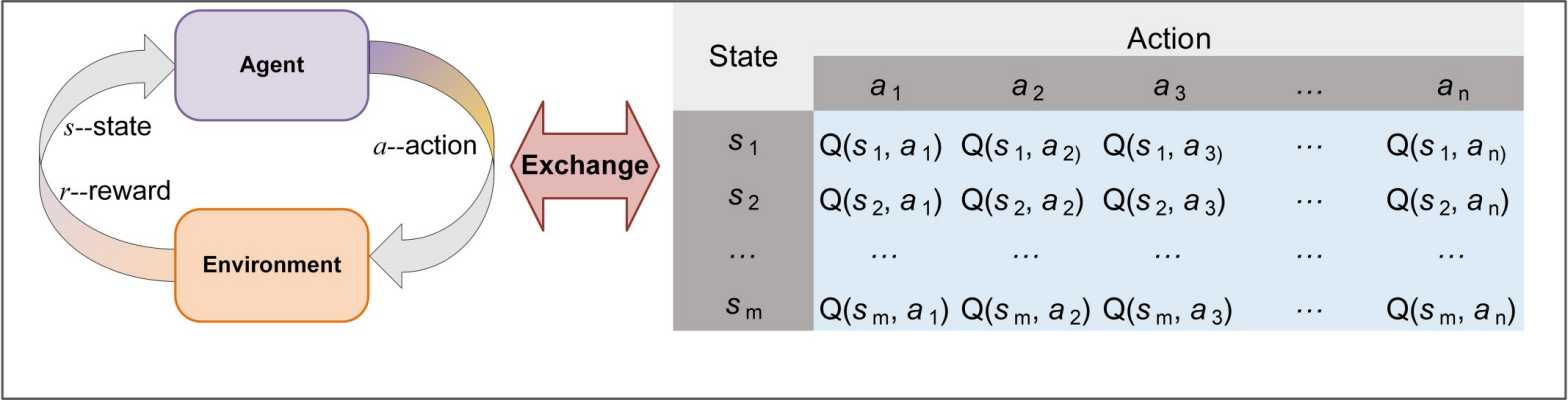
The *Q*-learning update process.

*Q*-learning [58] is a popular value-based reinforcement learning method that has numerous applications in areas such as supply chain management [59], time series forecasting [60], control systems [61], and computer games [62]. The *Q*-learning algorithm enables an agent to interact dynamically with its environment, allowing it to make specific actions based on the current state. This approach constructs a *Q*-table to select actions that can yield the maximum reward and continuously explores through an iterative process to discover potential global reward paths. As shown in Fig 4, *Q*-learning, like other reinforcement learning algorithms, consists of four primary components: the agent, states, rewards, and actions. After each interaction with the environment, the agent evaluates whether to change its state and whether to receive rewards. Subsequently, it updates the *Q*-table to adapt to the demands of the environment. Specifically, the update process of the *Q*-table can be expressed by the following formula:

$$Q(s_{t},a_{t})=Q(s_{t},a_{t})+\alpha [r_{t+1}+\gamma \max Q(s_{t+1},a^{\prime })-Q(s_{t},a_{t})]$$ (9)

where *α* represents the learning rate, which determines the extent to which new knowledge covers old knowledge, *α*∈[0,1]; *γ* represents the discount factor, which represents the importance of future rewards compared to immediate rewards, *γ*∈[0,1]; *r*_*t+*1_ represents the reward received at the next time step; *s*_*t+*1_ represents the state of the environment at the next time step; *a’* is the action that yields the highest *Q* value for a given state *s*_*t+*1_.

#### C. The proposed QLDE algorithm

The exploration of parameter settings for Differential Evolution (DE) has been ongoing for many years; however, the relationship between these parameters and performance remains inadequately defined. The fixed nature of the scaling factor *F* in traditional DE limits its ability to adapt to challenges encountered during the search process, such as stagnation. To address this issue, a hybrid approach that combines *Q*-learning with the Differential Evolution algorithm is proposed, enabling the algorithm to autonomously adapt the scaling factor without the need for predefined parameter settings throughout the search process.

In addition, traditional *Q*-learning often faces the risk of becoming trapped in local optima during the search process. To address this challenge and effectively integrate with the differential evolution algorithm, a dynamic ε-greedy strategy [63] is introduced to improve the expected selection process in *Q*-learning. By incorporating randomness, this approach helps prevent the agent from converging to a local optimum during the learning phase, thereby increasing the likelihood of discovering the global optimum. The updated process for the *Q*-table can be expressed as follows:

$$\exp\;Q=\{\begin{array}{l} \max\;Q(s_{t+1},a^{\prime }),\;if(rand<1-\varepsilon \cdot \frac{k}{k_{\max}})\\ \mathrm{rand}\_\mathrm{choice}(),\;else \end{array}$$ (10)

$$Q(s_{t},a_{t})=Q(s_{t},a_{t})+\alpha [r_{t+1}+\gamma \cdot \exp Q-Q(s_{t},a_{t})]$$ (11)

where *ε* is the greedy threshold, where a larger value indicates greater randomness. rand_choice() refers to randomly selecting an action from a predefined set of actions.

In the proposed method of QLDE, the probability-based Softmax strategy [64] is employed for action selection to determine the most likely action that *Q*-learning would execute in a specific state, as follows:

$$\pi (s_{i},a_{j})=\mathrm{Softmax}[Q(s_{i},a_{j})]=\frac{\exp[Q(s_{i},a_{j})]}{\sum_{j=1}^{D}\exp[Q(s_{i},a_{j})]}$$ (12)

where *π*(*s*_*i*_, *a*_*j*_) represents the probability of taking action *a*_*j*_ in state *s*_*i*_; *D* represents the total number of possible actions of the agent.

**Fig 5:**
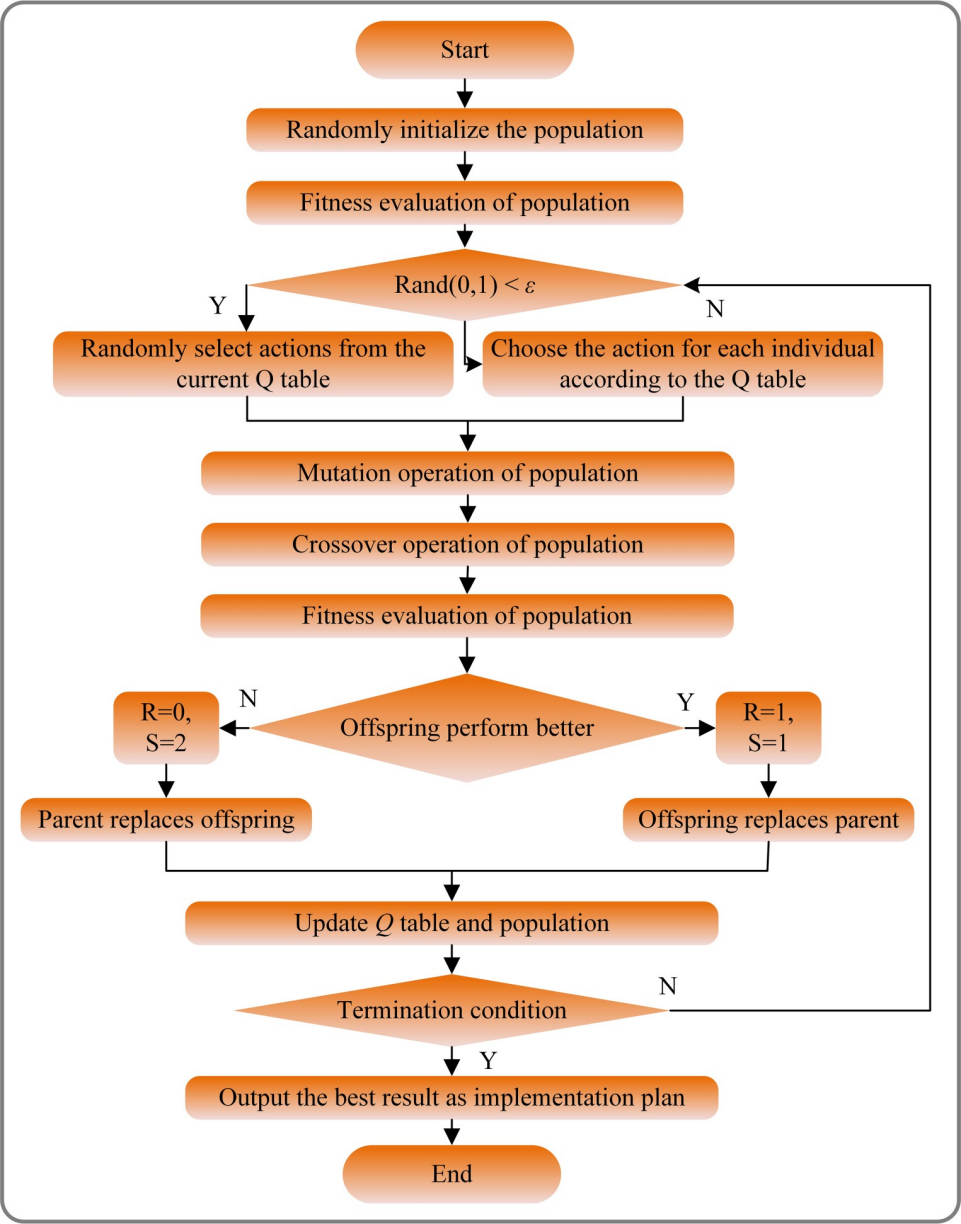
The flowchart of QLDE algorithm.

Finally, each individual is assigned a separate scaling factor, with three specified behaviors: *λ* = -0.01, *λ* = 0, and *λ* = 0.01, ensuring that the scaling factor can be adaptively adjusted. Furthermore, a reward of R = 1 and a state of S = 1 are granted to the agent only when the fitness of the trial individual exceeds that of the parent generation; otherwise, the reward is set to R = 0 and the state to S = 2. The detailed process of QLDE is shown in Fig 5. The adjustment method for the *i*-th individual through *Q*-learning can be expressed as follows:

$$F_{i}=F_{i}+\lambda_{i}$$ (13)

where *F*_*i*_ and *λ*_*i*_ are the scaling factor and adjustment factor corresponding to the *i*-th individual.

### 3.4 Customer segmentation based on *K*-means-QLDE

Although the *K*-means algorithm is simple and efficient, its accuracy is highly dependent on the selection of the initial cluster centers. Different initializations can lead to varying clustering results, and poorly chosen initial centers may even result in significantly inferior outcomes.

**Fig 6:**
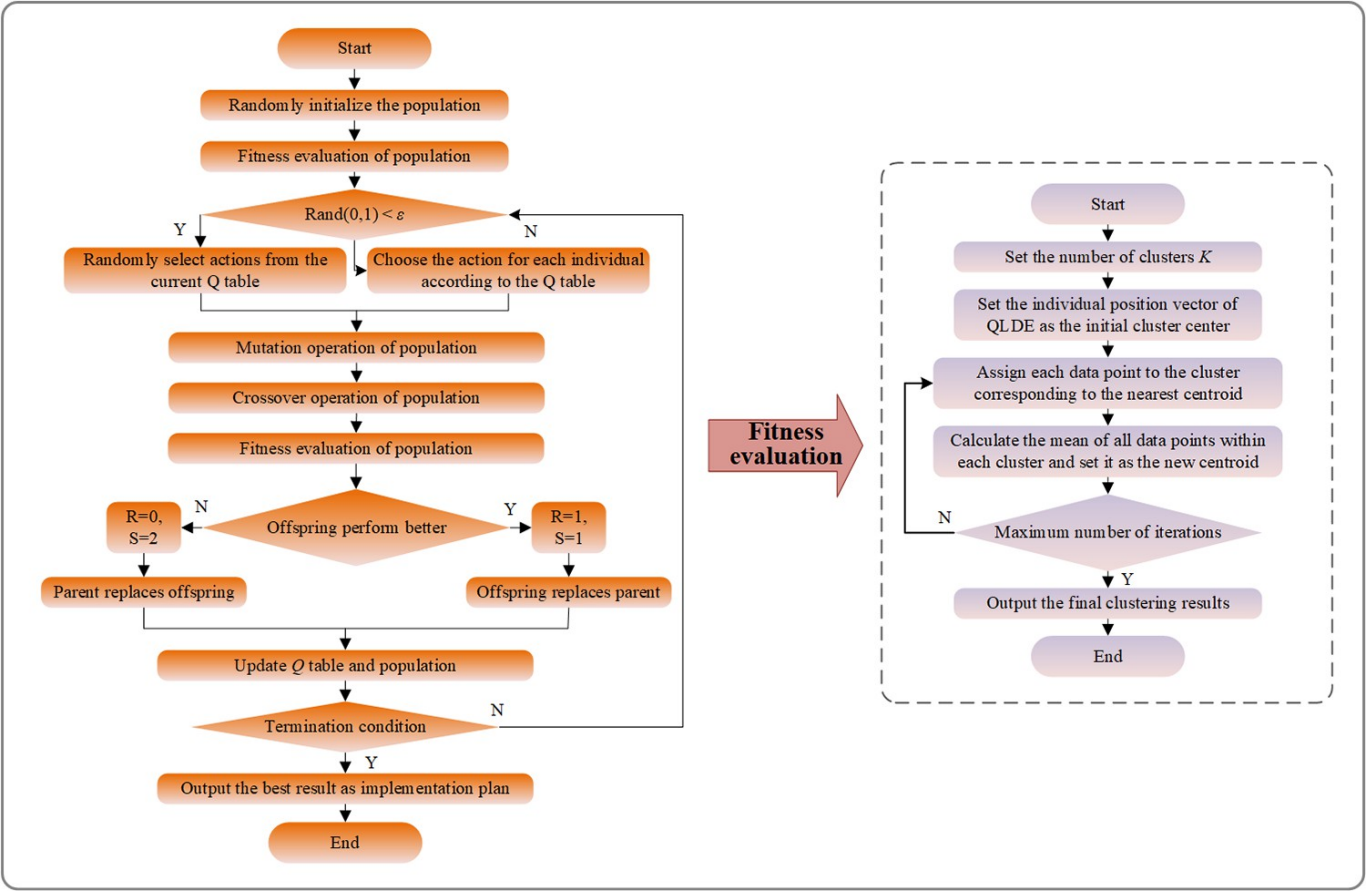
The implementation process of *K*-means-QLDE.

To address the challenge of setting initial cluster centers in the traditional *K*-means algorithm, we propose a *K*-means-QLDE method based on a dimensionality reduction approach. This method first employs PCA to linearly transform high-dimensional data into a lower-dimensional space, utilizing cumulative variance to select the principal components, which represent the features of the lower-dimensional space. Then, we combine the QLDE algorithm with *K*-means for adaptive clustering. Specifically, the number of clusters and the corresponding low-dimensional features serve as inputs to the QLDE algorithm, optimizing with the SSE of *K*-means as the objective to obtain the final distance results. The implementation process of *K*-means-QLDE is illustrated in Fig 6.

## 4. Results and discussions

To validate the effectiveness of the method, we used the highly credible Kaggle customer segmentation dataset [65] to verify the proposed digital marketing customer segmentation method. The original data consists of eight features, namely “InvoiceNo”, “StockCode”, “Description”, “Quantity”, “InvoiceDate”, “UnitPrice”, “CustomerID” and “Country”. To better capture the potential patterns and relationships within the data and enhance the predictive capability of our machine learning model, we employed the RFM (Recency, Frequency, Monetary) method to transform the original features into more meaningful information, as illustrated in Table 1.

**Table 1:** Description of the new features after transform.

No.	Feature	Description
**1**	Var 1	The number of days since last purchase of the customer
**2**	Var 2	Total number of transactions
**3**	Var 3	Total number of products by customer
**4**	Var 4	Total expenditure on purchased items
**5**	Var 5	Average transaction cost
**6**	Var 6	Number of product types purchased
**7**	Var 7	Average number of days to purchase
**8**	Var 8	Expected purchase days
**9**	Var 9	From the country of UK
**10**	Var 10	Transaction cancellation frequency
**11**	Var 11	Average monthly expenditure

### 4.1 Data preprocessing and feature dimension reduction

Data normalization is a crucial step in data preprocessing, aimed at bringing features of different dimensions onto the same scale. This process helps reduce the instability of numerical calculations and enhances model performance. In this paper, we employ Z-score normalization to process the transformed features. The formula for Z-score normalization [66] is expressed as follows:

$$X=\frac{X_{o}-\mu }{\sigma }$$ (14)

where ***X*** represents standardized dataset; ***X***_*o*_ represents the original dataset, while ***μ*** and ***σ*** represent the mean and standard deviation vectors for each feature in ***X***_*o*_, respectively.

**Fig 7:**
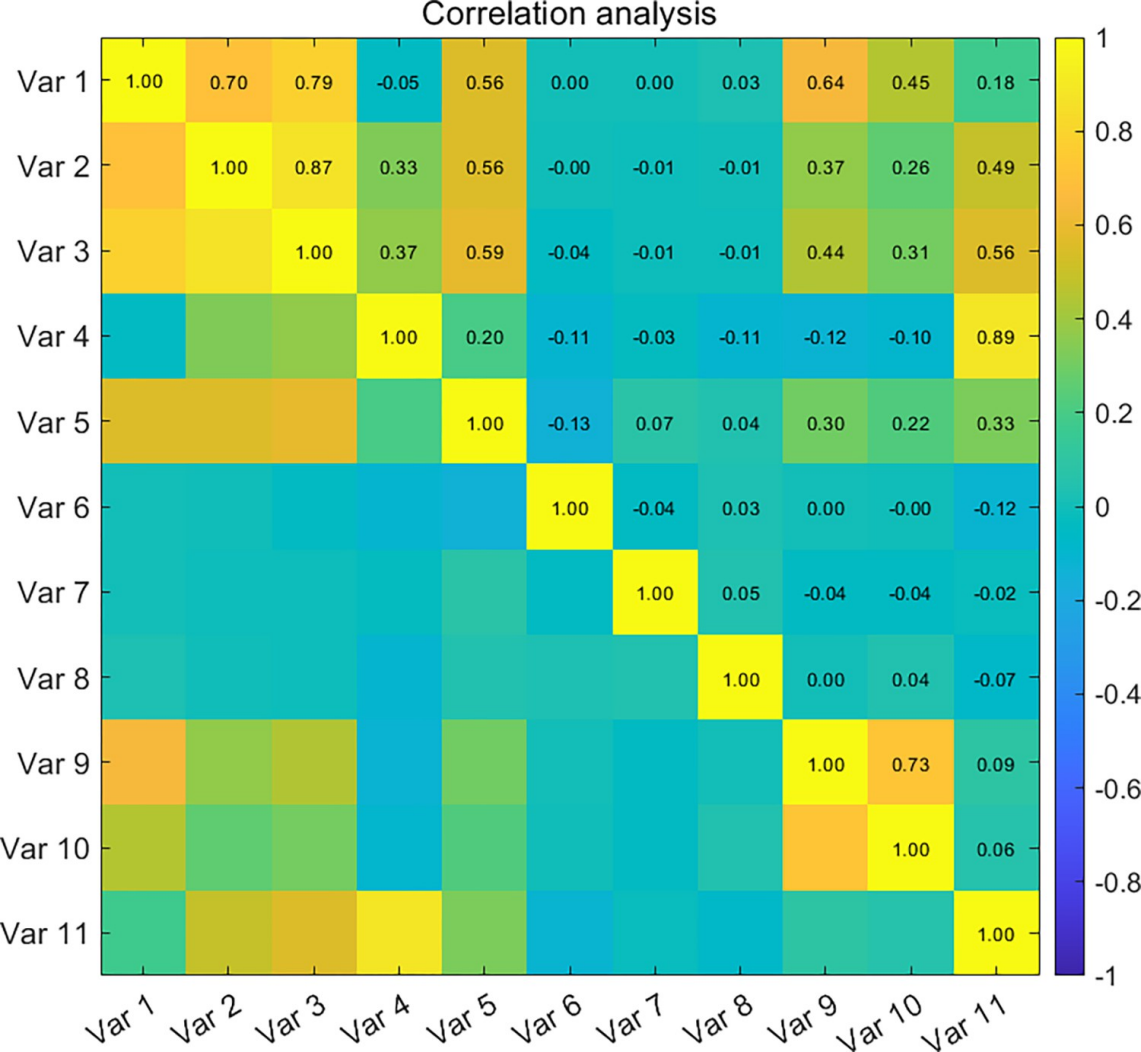
Correlations between different features.

To assess whether the transformed features provide distinct characteristic information, Fig 7 presents the results of the correlation analysis among various feature variables. In the figure, a darker yellow block indicates a stronger positive correlation, while a darker blue block signifies a stronger negative correlation. From the analysis, it is evident that there are significant correlations among the variables. For instance, feature var 1 exhibits a strong positive correlation with feature variables 2, 3, 5, and 9.

**Fig 8:**
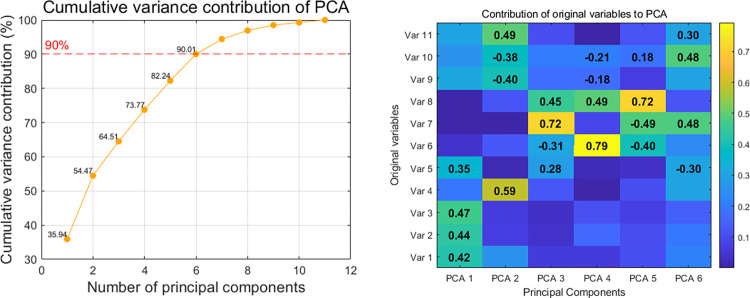
Principal component variance and features contribution. (a) Cumulative variance of PCA, (b) The contribution of the variables to PCA.

Taking into account the varying degrees of correlation between variables and aiming to enhance the quality of customer segmentation, Fig 8(A) and 8(B) illustrate the results of the different principal components obtained through the PCA method, including the cumulative explained variance contribution and the importance of the top four original features. From Fig 8(A), it is evident that as the number of principal components increases, the growth rate of cumulative explained variance gradually decreases. To ensure effective clustering and minimize data noise, the first 6 principal components, which account for more than 90% of the explained variance, are selected as input. Furthermore, Fig 8(B) demonstrates that after PCA processing, the principal components effectively represent the various characteristics of the original customers, confirming that this approach serves as an effective dimensionality reduction strategy.

### 4.2 Clustering applications of *K*-means-QLDE

**Fig 9:**
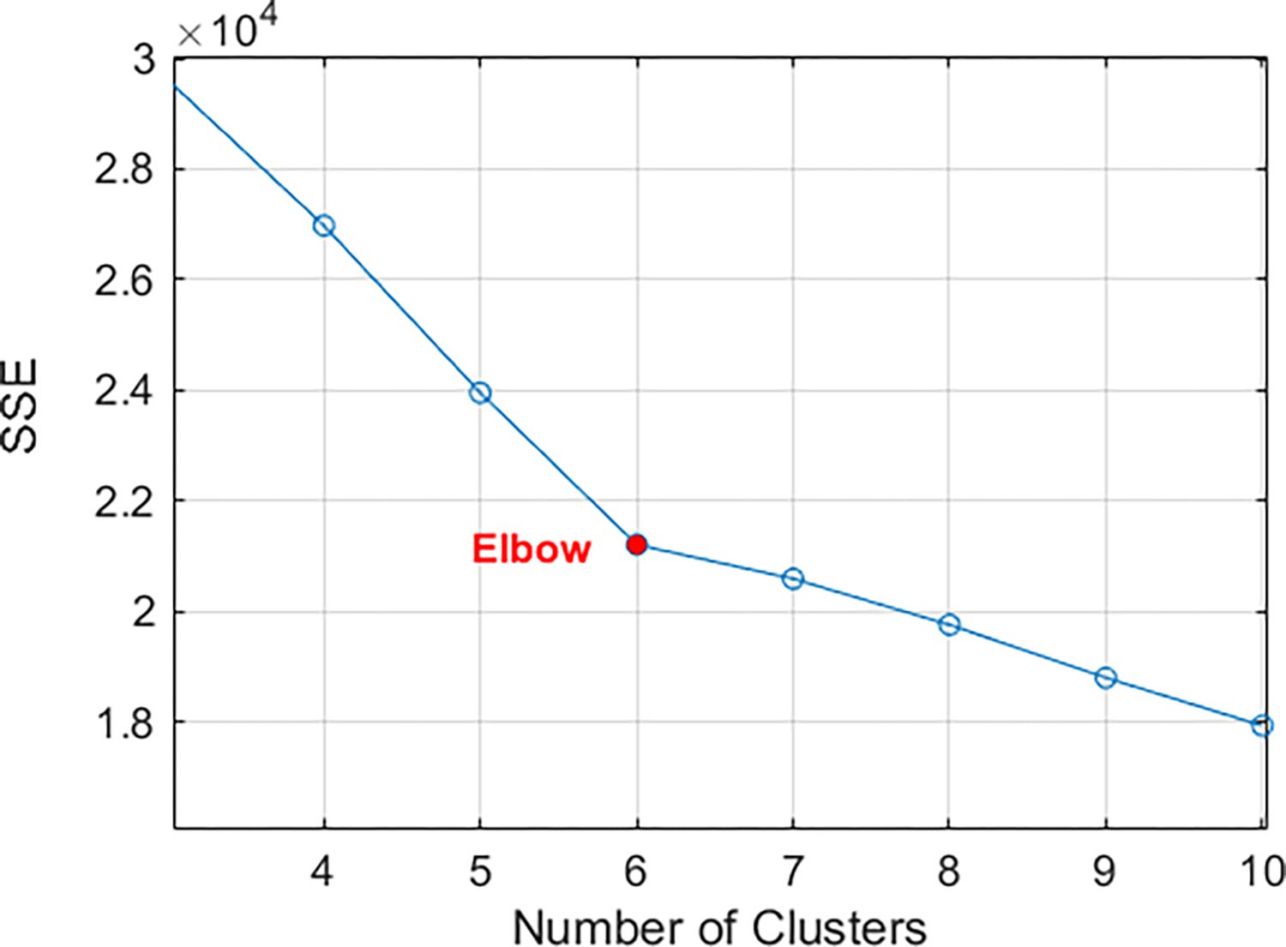
Relationship between the number of clusters and SSE.

To achieve effective clustering with the *K*-means algorithm, careful selection of the number of clusters *K* is essential. To this end, we employed the widely used elbow method [67], which identifies an “elbow” point as the optimal number of clusters by calculating the SSE for various values of *K*, as illustrated in Fig 9 From the figure, it is evident that as the value of *K* increases, the SSE decreases, indicating that customer segmentation data can achieve a lower SSE with more clusters. It is important to highlight that at *K* = 6, a clear inflection point indicates a significant improvement in the clustering performance of the *K*-means method, marking it as the optimal number of clusters for achieving the best results in our analysis.

**Fig 10:**
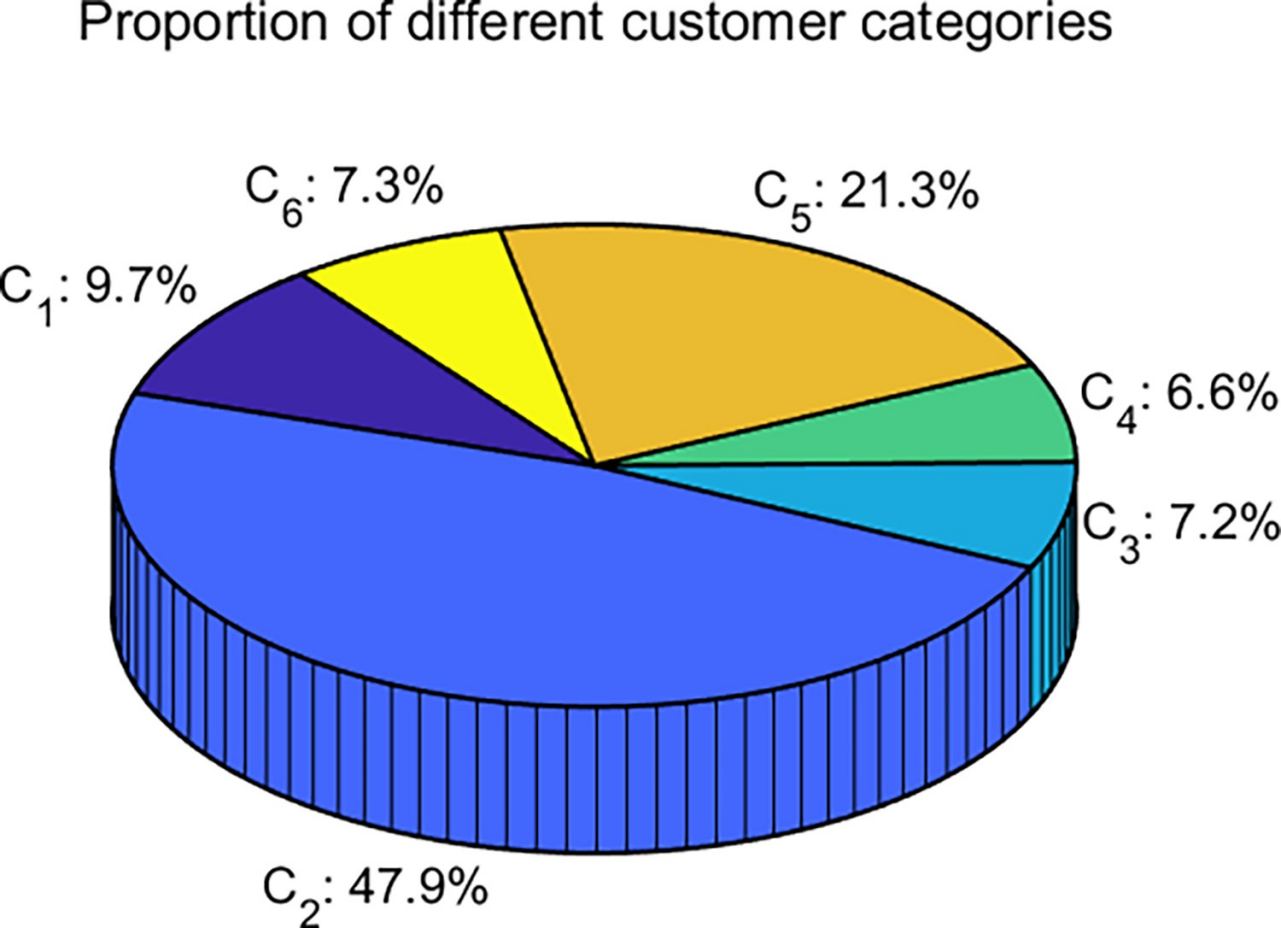
The proportion of different customer categories.

Fig 10 shows the segmentation results of different customer segments obtained through the *K*-means-QLDE algorithm. From the figure, it can be seen that Cluster 2 accommodates 47.9% of the customers, while Cluster 5 contains 21.3% of the customers. These two clusters account for the vast majority of all customers, allowing business managers can focus on analyzing the characteristics of this potential customer group and formulate corresponding business strategies. In the remaining clusters (1, 3, 4, and 6), the distribution of customers is relatively balanced. Although each cluster occupies a relatively small proportion, the overall level accounts for 30.8% of all customers. Therefore, it is essential to gain a deeper understanding of the characteristics of these customer groups to adapt to the needs of future business development.

**Fig 11:**
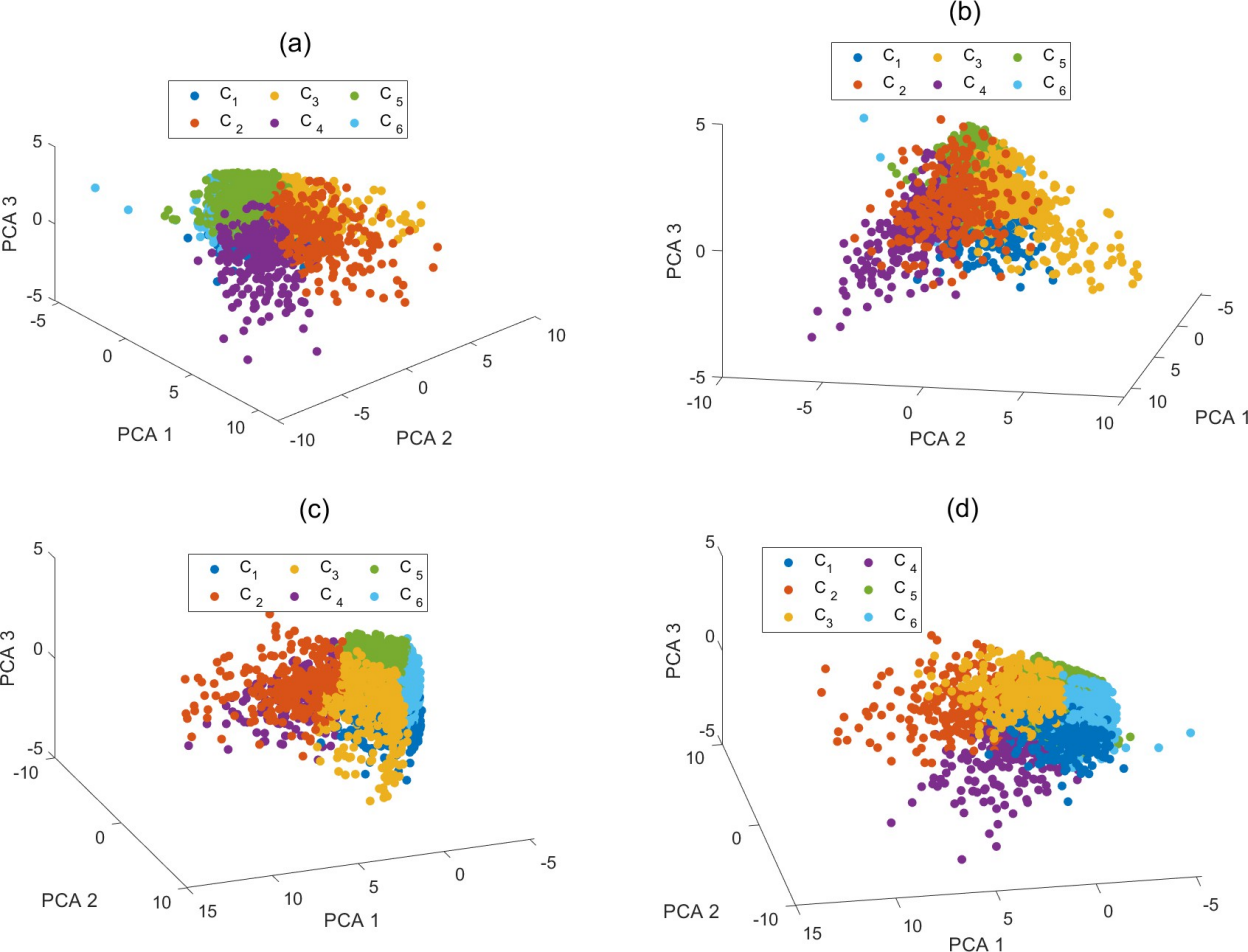
The clustering results in the first three principal components.

Fig 11 further illustrates the customer segmentation results obtained using the *K*-means-QLDE algorithm. The figure clearly demonstrates that different customers form distinct clusters in the space defined by the first three principal components. Each cluster represents a group of customers with similar characteristics, and there is no overlap between the groups in the principal component space, underscoring the effectiveness and validity of the clustering results. Furthermore, analyzing these clusters allows for the identification of behavioral patterns and demand characteristics among various customer segments, providing valuable data support for the development of subsequent marketing strategies.

**Fig 12:**
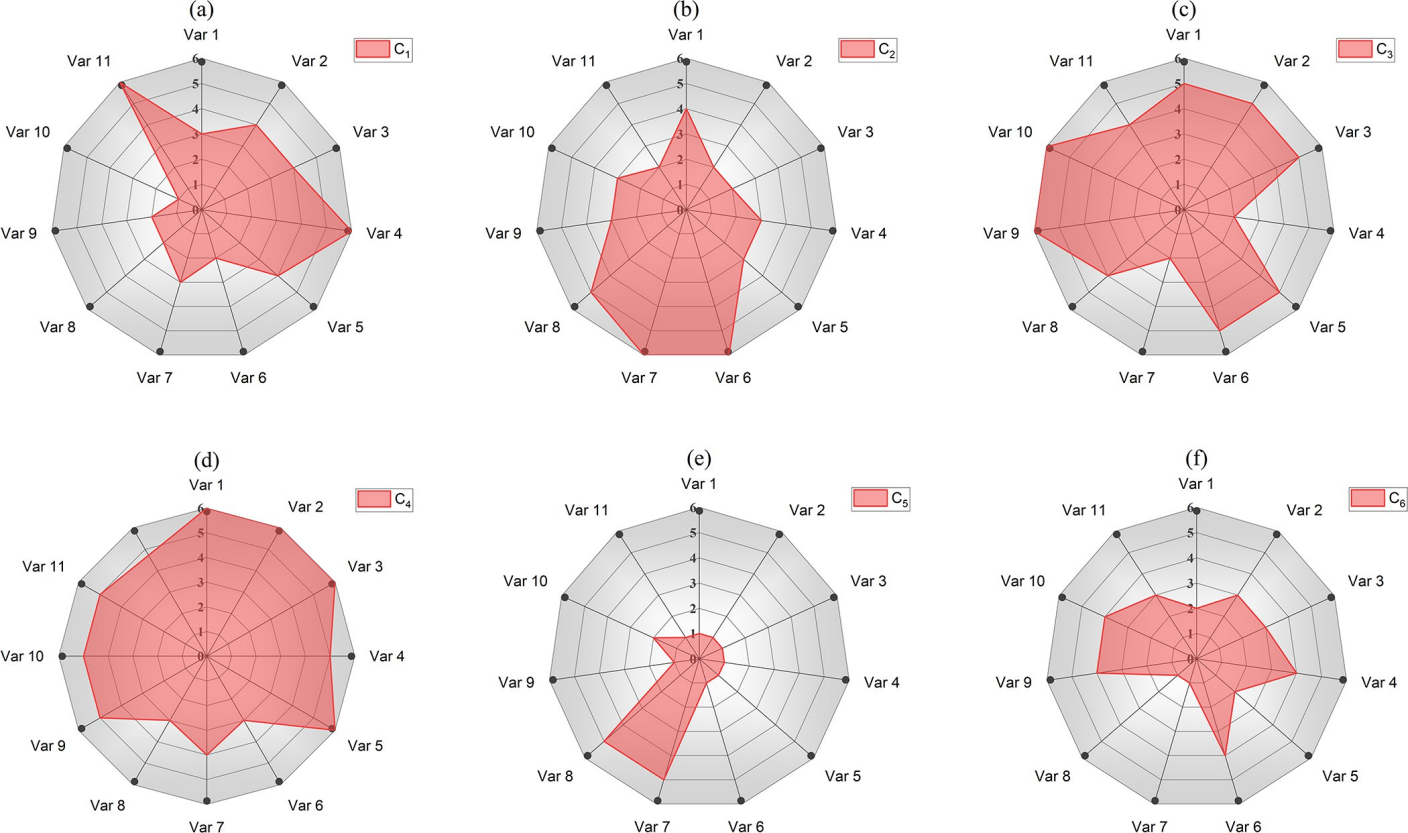
Ranking of cluster centers in different features.

Fig 12 illustrates the normalized statistical ranking results of the six cluster centers obtained through the 11 extracted features. This figure intuitively shows the differences between different groups in different dimensions. For example, for the customers in Fig 12(A), they have the characteristics of high average monthly expenditure, the most expenditure products, and low transaction cancellation frequency. Such customers are usually the most important customer groups for enterprises because they contribute the most to revenue and purchase frequently.

The customers represented in Fig 12(B) constitute 47.9% of the total customer base. They exhibit the highest volume of product purchases; however, they demonstrate a lower average purchase frequency and monthly expenditure. This suggests that they are inclined to buy low-priced or discounted items, or that they approach their purchasing decisions with greater rationality. Such customers can be categorized as price-sensitive consumers. To effectively engage this group, the industry should develop targeted marketing strategies aimed at stimulating their consumption, thereby fostering the long-term growth of the enterprise.

Fig 12(C) and 12(D) mainly illustrate the characteristics of high-expectation customers, especially focusing on the relationship between transaction cancellation rate and consumer origin, especially consumers from the UK. As can be seen from the figure, these consumers show a certain degree of uncertainty or indecision in their purchase decisions. In addition, British consumers may have higher expectations for product quality, which makes them more inclined to re-evaluate orders after purchase. Companies should consider implementing strategies to provide clearer product information and optimize the payment process to reduce transaction cancellation rates and improve customer satisfaction.

Fig 12(E) illustrates the characteristics of cautious consumers. Such customers exhibit a higher level of caution in making purchase decisions, often engaging in multiple comparisons and research. Their purchasing behavior is primarily concentrated on weekends, and they tend to be more rational and planned in their buying processes. Therefore, merchants can implement targeted marketing and promotional activities during the weekend. Fig 12(F) depicts the characteristics of balanced customers. These customers demonstrate relatively balanced performance across multiple purchasing indicators, without obvious preferences or extreme behaviors. Merchants can maintain and enhance customer relationships through diversified marketing strategies.

### 4.3 Classification accuracy verification based on machine learning

To further illustrate the practicality of the proposed refined customer segmentation method, the obtained clustering results were classified using several algorithms: Kernel Support Vector Machine (KSVM) [68], Decision Tree (DT) [69], AdaBoost [70], and Artificial Neural Network (ANN) [71]. 80% of the data was utilized as the training set, while 20% served for testing. Additionally, 5-fold cross-validation [72] was employed to adjust hyperparameters of the model during training, enhancing generalization ability and performance of the model. The first six principal components, derived from the input factors using the PCA method described in the previous section, were used as the input of the models.

**Fig 13:**
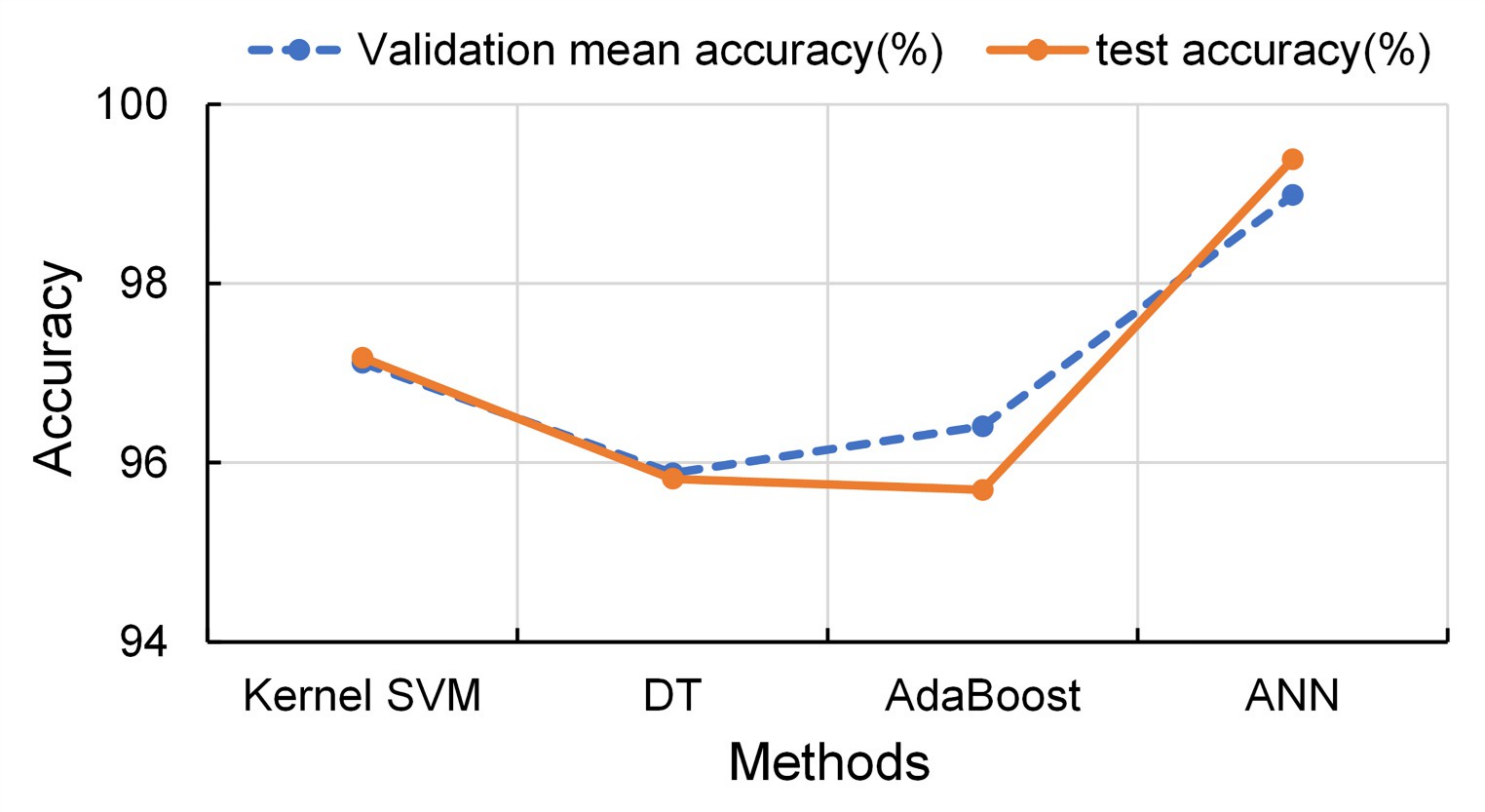
Classification accuracy of different methods.

The implementation was carried out using Statistics and Machine Learning Toolbox of MATLAB software. The analysis results are shown in Table 2, and the process of different classification accuracy is shown in Fig 13. The table and figure indicate that all methods achieved a classification accuracy exceeding 95% on the test set, demonstrating that the proposed customer segmentation method effectively ensures high classification accuracy. Notably, the ANN method achieved the highest classification accuracy, followed closely by KSVM. However, the time cost associated with the ANN is approximately 29 times greater than that of KSVM. Therefore, if merchants do not prioritize time efficiency, the ANN method may be preferred for customer classification; otherwise, KSVM is recommended. In summary, the proposed *K*-means-QLDE method effectively identifies key information among customers, significantly enhancing the marketing capabilities of merchants.

**Table 2:** Statistical results of classification using different methods.

Methods	Validation mean accuracy (%)	test set accuracy (%)	Time(Seconds)
**KSVM**	97.11	97.17	7.63
**DT**	95.88	95.82	10.15
**AdaBoost**	96.40	95.69	2.84
**ANN**	98.99	99.38	221.14

## 5. Conclusions and future works

This paper presents a novel method for customer segmentation in the digital marketing process, termed *K*-means-QLDE, which integrates heuristic algorithms with machine learning techniques. Initially, the PCA method is employed to extract principal component features from customer variables. Subsequently, a differential evolution algorithm based on *Q*-Learning (QLDE) is introduced to address the clustering problem, while the elbow method is utilized to ascertain the optimal number of clusters for the *K*-means algorithm. Utilizing a customer dataset sourced from Kaggle, the proposed method effectively segments customers into six distinct clusters, each representing varying levels of importance. To validate the practicality of this customer segmentation approach, four widely recognized methods are employed to assess the classification accuracy of the segmentation results. The findings indicate that the proposed method achieves a segmentation accuracy exceeding 95% on the test set. These results underscore the practical applicability of the *K*-means-QLDE segmentation method, empowering marketers to devise tailored digital marketing strategies for diverse customer groups, thereby enhancing the company’s marketing revenue.

Nevertheless, this study acknowledges several limitations. Firstly, while the proposed QLDE algorithm demonstrates effective clustering performance, it simultaneously complicates model interpretation. Secondly, the inherent complexity of the QLDE algorithm may present challenges when applied to large datasets. In future research, we aim to address these limitations by investigating more transparent dimensionality reduction techniques and optimization algorithms. Additionally, we plan to broaden the scope of our research to encompass high-dimensional datasets across various industries.
